# Supplementary material for: Empirical methods for controlling false positives and estimating confidence in ChIP-Seq peaks
Source: BMC Bioinformatics. 2008 Dec 5;9:523. doi: 10.1186/1471-2105-9-523 (PMC2628906; doi:10.1186/1471-2105-9-523)
Supplement: Additional File 1 — A variety of html documents from the USeq web site detailing the available applications, their best usage, output file type descriptions, command line menus, etc. [file 1471-2105-9-523-S1.zip › USeqUserGuides/JavaDoc.zip!/JavaDoc/util/gen/Misc.html]

Misc


|  |  |  |
| --- | --- | --- |
| **Overview** \| **Package** \| **Class** \| **Use** \| **Tree** \| **Deprecated** \| **Index** \| **Help** | |  |
| **PREV CLASS**   **NEXT CLASS** | **FRAMES**    **NO FRAMES**     **All Classes** |  |
| SUMMARY: NESTED \| FIELD \| CONSTR \| METHOD | DETAIL: FIELD \| CONSTR \| METHOD |  |


---


## util.gen Class Misc

```
java.lang.Object
  util.gen.Misc
```

---

``` public class Misc extends java.lang.Object ```

A variety of static methods.

---

**Constructor Summary**

`Misc()`


| **Method Summary** | |
| --- | --- |
| `static java.lang.String` | `addZeros(java.lang.String x)`             Prepends zeros to a string such that its total length = 10. |
| `static java.lang.String[]` | `addZeros(java.lang.String[] x)`             Prepends zeros to each String such that its total length = 10. |
| `static java.lang.String` | `capitalizeFirstLetter(java.lang.String s)`             Capitalizes the first letter in a String. |
| `static java.lang.String` | `clipUnderScore(java.lang.String string)`             Removes the random digit\_ proceeding a string, (ie 2432344322\_fileName.txt to fileName.txt) |
| `static int` | `countTrues(boolean[] b, int startIndex, int stopIndex)`             Does not check if indexes are out of bounds! |
| `static java.util.HashMap` | `createHashMap(java.lang.String[] s)`             Creates a HashMap from a String[] where the array is ordered such as key,value,key,value... |
| `static java.util.LinkedHashMap` | `createLinkedHashMap(java.lang.String[] s, int direction)`             Creates a LinkedHashMap from a String[] where the array is ordered such as key,value,key,value... |
| `static java.lang.String` | `doubleArrayToString(double[] i, java.lang.String separator)`             Returns a String separated by separator for each double[i]. |
| `static int` | `extractIdFromConcat(java.lang.String concat)`             Takes a String like testSlide:33328:testslide desc...:1 or test: gip: 2, splits on the : and looks for ID in the last String[x], if not found it returns zero, otherwise it returns a parsed int. |
| `static int` | `extractIdFromFrontOfConcat(java.lang.String concat)`             Takes a String like 1: testSlide: 33328:testslide desc etc or 2: test: gip, splits on the : and looks for ID in the first String[x], if not found it returns zero, otherwise it returns a parsed int. |
| `static java.lang.String[]` | `extractIDsFromConcats(java.lang.String[] concats)`             Ditto to extractIDFromConcat but works on String[] returning IDs as a String[]. |
| `static java.lang.String` | `fetchExit(java.lang.String key, java.util.HashMap map)`             Looks for key, if not found prints error message and exists. |
| `static int` | `fetchMatchingStringArrayIndex(java.lang.String guess, java.lang.String[] choices)`             Loose matching of substring guess from larger String[]s. |
| `static java.lang.String` | `floatArrayToString(float[] i, java.lang.String separator)`             Returns a String separated by separator for each float[i]. |
| `static java.lang.String` | `getDate()`             Returns a nicely formated time (15 May 2004). |
| `static java.lang.String` | `getDateNoSpaces()`             Returns a nicely formated time (15May2004). |
| `static java.lang.String` | `getFormattedTimeFromFraction(double fractionalTime)`             Returns a formatted time 2:15:00 given 2.25 |
| `static java.lang.String[]` | `hashSetToStringArray(java.util.HashSet hash)`             Converts a hash to a String[] by direct cast. |
| `static java.lang.String` | `intArrayToString(int[] i, java.lang.String separator)`             Returns a String separated by separator for each int[i]. |
| `static int[]` | `integerArrayListToIntArray(java.util.ArrayList integerAL)`             Converts an ArrayList of Integer to int[]. |
| `static java.util.HashMap` | `invert(java.util.HashMap h)`             Flips key:value to value:key. |
| `static boolean` | `isEmpty(java.lang.String text)`             Method to check if a string is null or empty after trimming. |
| `static boolean` | `isNotEmpty(java.lang.String text)`             Method to check if a string is not null and contains more than "". |
| `static boolean` | `isStringEmpty(java.lang.String string)`             Returns true if a String contains just white space or is empty. |
| `static java.util.HashSet` | `loadHashSet(java.lang.Object[] s)`             Loads a HashSet with and Object array. |
| `static java.util.ArrayList` | `objectArrayToArrayList(java.lang.Object[] ob)`             Converts an Object[] to an ArrayList |
| `static java.util.ArrayList` | `parseKeyValueGroups(java.lang.String line)`             Given a String of 'treat1=/file1,/file2\*treat2=/file3,/file4\*treat3=/file5,file6' Will return an ArrayList contining first a String[] of the keys {treat1,treat2,treat3} and second a String[][] of the parsed values matching the keys. |
| `static java.util.LinkedHashMap` | `parseKeyValues(java.lang.String line)`             Given a String of 'treat1=/file1,treat2=/file3, etc', no spaces, returns a LinkedHashMap of keys=values. |
| `static java.lang.String[]` | `prependString(java.lang.String[] d, java.lang.String newValue)`             Returns a new array with the newValue appended on the beginning. |
| `static void` | `printArray(java.util.ArrayList array)`             Prints ArrayList to System.out |
| `static void` | `printArray(boolean[] array)`             Prints a boolean[] to System.out |
| `static void` | `printArray(byte[] array)`             Prints a byte[] to System.out |
| `static void` | `printArray(double[] array)`             Prints a double[] to System.out on a single line. |
| `static void` | `printArray(double[][] d)`             Print to System.out a double[][] |
| `static void` | `printArray(float[] array)`             Prints a int[] to System.out |
| `static void` | `printArray(float[][] d)`             Print to System.out a float[][] |
| `static void` | `printArray(int[] array)`             Prints a int[] to System.out |
| `static void` | `printArray(int[][] d)`             Print to System.out a int[][] |
| `static void` | `printArray(java.lang.Object[] array)`             Prints a Object[] to System.out |
| `static void` | `printArray(java.lang.Object[][] array)`             Prints a Object[][] to System.out |
| `static void` | `printArray(java.lang.String[] array)`             Prints a String[] to System.out |
| `static void` | `printArrayLine(java.lang.String[] array)`             Prints a String[] to System.out |
| `static void` | `printErrAndExit(java.lang.String message)`             Prints message to screen, then exits. |
| `static void` | `printExit(java.lang.String message)`             Prints message to screen, then exits. |
| `static void` | `printHashMap(java.util.HashMap hash, java.lang.String seperator)`             Prints key + seperator + value+ return to out. |
| `static boolean` | `printHashSetToFile(java.util.HashSet hash, java.lang.String file)`             Prints a HashSet to a file. |
| `static void` | `randomize(java.lang.Object[] f, long seed)`             Randomizes the array by permutating indexes and swapping. |
| `static void` | `randomize(java.lang.String[] f, long seed)`             Randomizes the array by permutating indexes and swapping. |
| `static java.lang.String` | `removeExtension(java.lang.String txt)`             Removes an extension if found xxx.txt -> xxx. |
| `static java.lang.String` | `replaceEnd(java.lang.String word, java.lang.String match, java.lang.String replacement)`             Attempts to match the end of the word with the match, case insensitive, replaces it if found, otherwise returns null. |
| `static java.lang.String` | `reverse(java.lang.String x)`             Reverses a String using a StringBuilder. |
| `static void` | `sleep(int seconds)`             Sleeps for given number of seconds |
| `static java.lang.String[]` | `splitOnFirstColon(java.lang.String idColonName)`             Returns a String[2] split on first colon, if no colon returns null. |
| `static java.lang.String[]` | `splitString(java.lang.String concat, java.lang.String regex)`             Splits a String on a regular expression, watching out for escaped characters ie Cat\=Mouse would not be split when = is given as the regex, a String[0], {"Cat\=Mouse"} would be returned. |
| `static java.lang.String` | `stringArrayListToString(java.util.ArrayList stringAL, java.lang.String separator)`             Returns a String separated by the separator given an ArrayList of String. |
| `static java.lang.String[]` | `stringArrayListToStringArray(java.util.ArrayList stringAL)`             Returns a String[] given an ArrayList of Strings. |
| `static java.util.ArrayList` | `stringArrayToArrayList(java.lang.String[] s)`             Returns an ArrayList of String given a String[] |
| `static java.lang.String` | `stringArrayToString(java.lang.String[] s, java.lang.String separator)`             Returns a String separated by commas for each bin. |
| `java.lang.String[]` | `toLowerCase(java.lang.String[] strings)`             Lower cases a String[] |
| `static int` | `totalLength(java.lang.Object[][] array)`             Counts the total length of an Object[][] |
| `static void` | `trim(java.lang.String[] s)`             Trims all the strings in the array String.trim() |
| `static java.lang.String[]` | `trimCommon(java.lang.String[] lines)`             Trims the comon chars from the front and back of each line, won't entirely delete line. |
| `static java.lang.String[]` | `trimCommonStart(java.lang.String[] lines)`             Trims characters common to all from start of lines. |
| `static java.lang.String` | `trimQuotes(java.lang.String cell)`             Trims "" from the beginning and end of String if both are found. |
| `static java.lang.String` | `trimTxt(java.lang.String s)`             Removes .txt from end of String if present |
| `static java.lang.String` | `truncate(java.lang.String string, int cutOff)`             Truncates a String adding three periods if too long. |

**Methods inherited from class java.lang.Object**

`equals, getClass, hashCode, notify, notifyAll, toString, wait, wait, wait`

**Constructor Detail**

### Misc

```
public Misc()
```


**Method Detail**

### invert

```
public static java.util.HashMap invert(java.util.HashMap h)
```

:   Flips key:value to value:key.

---


### reverse

```
public static java.lang.String reverse(java.lang.String x)
```

:   Reverses a String using a StringBuilder.

---


### randomize

```
public static void randomize(java.lang.String[] f,
                             long seed)
```

:   Randomizes the array by permutating indexes and swapping. Fast.
    Can set seedMultiplier or vary it to be sure and get diff

---


### randomize

```
public static void randomize(java.lang.Object[] f,
                             long seed)
```

:   Randomizes the array by permutating indexes and swapping. Fast.
    Can set seed or vary it to be sure and get diff

---


### countTrues

```
public static int countTrues(boolean[] b,
                             int startIndex,
                             int stopIndex)
```

:   Does not check if indexes are out of bounds!

    :   **Parameters:**: `startIndex` - is included: `stopIndex` - is excluded **Returns:**: the number of trues between the start and stop

---


### printHashMap

```
public static void printHashMap(java.util.HashMap hash,
                                java.lang.String seperator)
```

:   Prints key + seperator + value+ return to out.

---


### hashSetToStringArray

```
public static java.lang.String[] hashSetToStringArray(java.util.HashSet hash)
```

:   Converts a hash to a String[] by direct cast.

---


### sleep

```
public static void sleep(int seconds)
```

:   Sleeps for given number of seconds

---


### fetchExit

```
public static java.lang.String fetchExit(java.lang.String key,
                                         java.util.HashMap map)
```

:   Looks for key, if not found prints error message and exists.

---


### prependString

```
public static java.lang.String[] prependString(java.lang.String[] d,
                                               java.lang.String newValue)
```

:   Returns a new array with the newValue appended on the beginning.

---


### capitalizeFirstLetter

```
public static java.lang.String capitalizeFirstLetter(java.lang.String s)
```

:   Capitalizes the first letter in a String.

---


### removeExtension

```
public static java.lang.String removeExtension(java.lang.String txt)
```

:   Removes an extension if found xxx.txt -> xxx.
    If none found returns the original.

---


### parseKeyValueGroups

```
public static java.util.ArrayList parseKeyValueGroups(java.lang.String line)
```

:   Given a String of 'treat1=/file1,/file2\*treat2=/file3,/file4\*treat3=/file5,file6'
    Will return an ArrayList contining first a String[] of the keys {treat1,treat2,treat3}
    and second a String[][] of the parsed values matching the keys.
    Returns null if an error is found.

---


### trimCommonStart

```
public static java.lang.String[] trimCommonStart(java.lang.String[] lines)
```

:   Trims characters common to all from start of lines.

---


### trimCommon

```
public static java.lang.String[] trimCommon(java.lang.String[] lines)
```

:   Trims the comon chars from the front and back of each line, won't entirely delete line.

---


### parseKeyValues

```
public static java.util.LinkedHashMap parseKeyValues(java.lang.String line)
```

:   Given a String of 'treat1=/file1,treat2=/file3, etc', no spaces,
    returns a LinkedHashMap of keys=values.
    Returns null if an error is found.

---


### replaceEnd

```
public static java.lang.String replaceEnd(java.lang.String word,
                                          java.lang.String match,
                                          java.lang.String replacement)
```

:   Attempts to match the end of the word with the match, case insensitive, replaces it if found,
    otherwise returns null.

---


### printErrAndExit

```
public static void printErrAndExit(java.lang.String message)
```

:   Prints message to screen, then exits.

---


### printExit

```
public static void printExit(java.lang.String message)
```

:   Prints message to screen, then exits.

---


### totalLength

```
public static int totalLength(java.lang.Object[][] array)
```

:   Counts the total length of an Object[][]

---


### trimTxt

```
public static java.lang.String trimTxt(java.lang.String s)
```

:   Removes .txt from end of String if present

---


### addZeros

```
public static java.lang.String addZeros(java.lang.String x)
```

:   Prepends zeros to a string such that its total length = 10. Use for converting a String number to a form that can be
    correctly sorted. ie would convert 39 to 0000000039

---


### addZeros

```
public static java.lang.String[] addZeros(java.lang.String[] x)
```

:   Prepends zeros to each String such that its total length = 10. Use for converting a String number to a form that can be
    correctly sorted. ie would convert 39 to 0000000039

---


### loadHashSet

```
public static java.util.HashSet loadHashSet(java.lang.Object[] s)
```

:   Loads a HashSet with and Object array.

---


### trimQuotes

```
public static java.lang.String trimQuotes(java.lang.String cell)
```

:   Trims "" from the beginning and end of String if both are found.
    Excel when saving as a tab delimited file, adds quotes !$#%#$!!!

---


### splitString

```
public static java.lang.String[] splitString(java.lang.String concat,
                                             java.lang.String regex)
```

:   Splits a String on a regular expression, watching out for escaped characters
    ie Cat\=Mouse would not be split when = is given as the regex, a String[0],
    {"Cat\=Mouse"} would be returned. The method will not
    return empty Strings, Cat===Mouse would return {"Cat","Mouse}. Lastly,
    each String in the String[] is .trim()ed prior to saving, so no flanking
    white space. Use this method when you need to allow for character escaping.

---


### fetchMatchingStringArrayIndex

```
public static int fetchMatchingStringArrayIndex(java.lang.String guess,
                                                java.lang.String[] choices)
```

:   Loose matching of substring guess from larger String[]s.
    Returns index of first matching choice, -1 if nothing found.
    Case insensitive.

---


### toLowerCase

```
public java.lang.String[] toLowerCase(java.lang.String[] strings)
```

:   Lower cases a String[]

---


### printArray

```
public static void printArray(java.lang.Object[] array)
```

:   Prints a Object[] to System.out

---


### printArray

```
public static void printArray(byte[] array)
```

:   Prints a byte[] to System.out

---


### printArray

```
public static void printArray(boolean[] array)
```

:   Prints a boolean[] to System.out

---


### printArray

```
public static void printArray(java.lang.Object[][] array)
```

:   Prints a Object[][] to System.out

---


### printArray

```
public static void printArray(java.lang.String[] array)
```

:   Prints a String[] to System.out

---


### printArrayLine

```
public static void printArrayLine(java.lang.String[] array)
```

:   Prints a String[] to System.out

---


### printArray

```
public static void printArray(java.util.ArrayList array)
```

:   Prints ArrayList to System.out

---


### printArray

```
public static void printArray(int[] array)
```

:   Prints a int[] to System.out

---


### printArray

```
public static void printArray(float[] array)
```

:   Prints a int[] to System.out

---


### printArray

```
public static void printArray(double[] array)
```

:   Prints a double[] to System.out on a single line.

---


### trim

```
public static void trim(java.lang.String[] s)
```

:   Trims all the strings in the array String.trim()

---


### clipUnderScore

```
public static java.lang.String clipUnderScore(java.lang.String string)
```

:   Removes the random digit\_ proceeding a string, (ie 2432344322\_fileName.txt to fileName.txt)

---


### truncate

```
public static java.lang.String truncate(java.lang.String string,
                                        int cutOff)
```

:   Truncates a String adding three periods if too long.

---


### getDate

```
public static java.lang.String getDate()
```

:   Returns a nicely formated time (15 May 2004).
    (Note, all I can say is that the GC DateFormat Date classes are so convoluted as to be utterly useless. Shame!)

---


### getDateNoSpaces

```
public static java.lang.String getDateNoSpaces()
```

:   Returns a nicely formated time (15May2004).
    (Note, all I can say is that the GC DateFormat Date classes are so convoluted as to be utterly useless. Shame!)

---


### getFormattedTimeFromFraction

```
public static java.lang.String getFormattedTimeFromFraction(double fractionalTime)
```

:   Returns a formatted time 2:15:00 given 2.25

---


### extractIdFromConcat

```
public static int extractIdFromConcat(java.lang.String concat)
```

:   Takes a String like testSlide:33328:testslide desc...:1 or test: gip: 2,
    splits on the : and looks for ID in the last String[x], if not found it returns zero, otherwise
    it returns a parsed int.

---


### extractIdFromFrontOfConcat

```
public static int extractIdFromFrontOfConcat(java.lang.String concat)
```

:   Takes a String like 1: testSlide: 33328:testslide desc etc or 2: test: gip,
    splits on the : and looks for ID in the first String[x], if not found it returns zero, otherwise
    it returns a parsed int.

---


### extractIDsFromConcats

```
public static java.lang.String[] extractIDsFromConcats(java.lang.String[] concats)
```

:   Ditto to extractIDFromConcat but works on String[] returning IDs as a String[].
    Pulls off an int from the first cel of a colon delimited String.

---


### splitOnFirstColon

```
public static java.lang.String[] splitOnFirstColon(java.lang.String idColonName)
```

:   Returns a String[2] split on first colon, if no colon returns null.

---


### isEmpty

```
public static boolean isEmpty(java.lang.String text)
```

:   Method to check if a string is null or empty after trimming.

---


### isNotEmpty

```
public static boolean isNotEmpty(java.lang.String text)
```

:   Method to check if a string is not null and contains more than "".

---


### integerArrayListToIntArray

```
public static int[] integerArrayListToIntArray(java.util.ArrayList integerAL)
```

:   Converts an ArrayList of Integer to int[]. Returns null if empty.

---


### stringArrayListToStringArray

```
public static java.lang.String[] stringArrayListToStringArray(java.util.ArrayList stringAL)
```

:   Returns a String[] given an ArrayList of Strings.

---


### intArrayToString

```
public static java.lang.String intArrayToString(int[] i,
                                                java.lang.String separator)
```

:   Returns a String separated by separator for each int[i].
    Returns "" if null or uninitialized.

---


### floatArrayToString

```
public static java.lang.String floatArrayToString(float[] i,
                                                  java.lang.String separator)
```

:   Returns a String separated by separator for each float[i].
    Returns "" if null or uninitialized.

---


### doubleArrayToString

```
public static java.lang.String doubleArrayToString(double[] i,
                                                   java.lang.String separator)
```

:   Returns a String separated by separator for each double[i].
    Returns "" if null or uninitialized.

---


### createLinkedHashMap

```
public static java.util.LinkedHashMap createLinkedHashMap(java.lang.String[] s,
                                                          int direction)
```

:   Creates a LinkedHashMap from a String[] where the array is ordered such as key,value,key,value...
    Direction 0 forward, 1 = reverse of making the Map. Returns null if odd number of Strings

---


### printHashSetToFile

```
public static boolean printHashSetToFile(java.util.HashSet hash,
                                         java.lang.String file)
```

:   Prints a HashSet to a file. Each entry on a new line.

---


### objectArrayToArrayList

```
public static java.util.ArrayList objectArrayToArrayList(java.lang.Object[] ob)
```

:   Converts an Object[] to an ArrayList

---


### printArray

```
public static void printArray(double[][] d)
```

:   Print to System.out a double[][]

---


### printArray

```
public static void printArray(float[][] d)
```

:   Print to System.out a float[][]

---


### printArray

```
public static void printArray(int[][] d)
```

:   Print to System.out a int[][]

---


### stringArrayToArrayList

```
public static java.util.ArrayList stringArrayToArrayList(java.lang.String[] s)
```

:   Returns an ArrayList of String given a String[]

---


### stringArrayListToString

```
public static java.lang.String stringArrayListToString(java.util.ArrayList stringAL,
                                                       java.lang.String separator)
```

:   Returns a String separated by the separator given an ArrayList of String.

---


### stringArrayToString

```
public static java.lang.String stringArrayToString(java.lang.String[] s,
                                                   java.lang.String separator)
```

:   Returns a String separated by commas for each bin.

---


### createHashMap

```
public static java.util.HashMap createHashMap(java.lang.String[] s)
```

:   Creates a HashMap from a String[] where the array is ordered such as key,value,key,value...
    Returns null if odd number of Strings

---


### isStringEmpty

```
public static boolean isStringEmpty(java.lang.String string)
```

:   Returns true if a String contains just white space or is empty.


---


|  |  |  |
| --- | --- | --- |
| **Overview** \| **Package** \| **Class** \| **Use** \| **Tree** \| **Deprecated** \| **Index** \| **Help** | |  |
| **PREV CLASS**   **NEXT CLASS** | **FRAMES**    **NO FRAMES**     **All Classes** |  |
| SUMMARY: NESTED \| FIELD \| CONSTR \| METHOD | DETAIL: FIELD \| CONSTR \| METHOD |  |


---
